# 

**Published:** 1878-07

**Authors:** 


SURG. GEN'L'S OFF'S

Vol. XIV. JULY, 1878. No. 2.

### THE BISTOURY:

### A QUARTERLY JOURNAL,

*Devoted to the Exposition of Charlatanism in Medicine and the Education of the People upon Medical Subjects!*

Thad S. Up de Graff, M. D., Editor.

***Terms of Subscription, Fifty Cents per Annum, in Advance.***

ELMIRA, N. Y.

PRINTED FOR THE PROPRIETOR, AT THE DAILY ADVERTISER JOB PRINTING HOUSE.

